# Mechanical and Physicochemical Properties of Newly Formed ZnO-PMMA Nanocomposites for Denture Bases

**DOI:** 10.3390/nano8050305

**Published:** 2018-05-06

**Authors:** Mariusz Cierech, Izabela Osica, Adam Kolenda, Jacek Wojnarowicz, Dariusz Szmigiel, Witold Łojkowski, Krzysztof Kurzydłowski, Katsuhiko Ariga, Elżbieta Mierzwińska-Nastalska

**Affiliations:** 1 Department of Prosthodontics, Medical University of Warsaw, 02-006 Warsaw, Poland; adam.kolenda@poczta.fm (A.K.); elzbieta.mierzwinska-nastalska@wum.edu.pl (E.M.-N.); 2 Faculty of Materials Science and Engineering, Warsaw University of Technology, 02-504 Warsaw, Poland; izabela.osica@gmail.com (I.O.); Krzysztof.kurzydlowski@pw.edu.pl (K.K.); 3 World Premier International Center for Materials Nanoarchitectonics (WPI-MANA), National Institute for Materials Science (NIMS), 1-1 Namiki, Tsukuba 305-0044, Japan; ARIGA.Katsuhiko@nims.go.jp; 4 Institute of High Pressure Physics, Polish Academy of Sciences, 01-142 Warsaw, Poland; jacek.wojnarowicz@tlen.pl (J.W.); w.lojkowski@labnano.pl (W.Ł.); 5 Division of Silicon Microsystem and Nanostructure Technology, Institute of Electron Technology, 02-668 Warsaw, Poland; szmigiel@ite.waw.pl

**Keywords:** denture stomatitis, polymethyl methacrylate, zinc oxide nanoparticles, *Candida albicans*

## Abstract

Aim: The aim of this study was to investigate the selected properties of zinc oxide- polymethyl methacrylate (ZnO-PMMA) nanocomposites that can influence the microorganism deposition on their surface. Materials and Methods: Non-commercial ZnO-NPs were prepared, characterized and used for the preparation of PMMA nanocomposite. Roughness, absorbability, contact angle and hardness of this new nanomaterial were evaluated. PMMA without ZnO-NPs served as control. Outcomes: Compared to unenriched PMMA, incorporation of ZnO-NPs to 7.5% for PMMA nanocomposite increases the hardness (by 5.92%) and the hydrophilicity. After modification of the material with zinc oxide nanoparticles the roughness parameter did not change. All tested materials showed absorption within the range of 1.82 to 2.03%, which meets the requirements of International Organization for Standardization (ISO) standards for denture base polymers. Conclusions: The results showed no significant deterioration in the properties of acrylic resin that could disqualify the nanocomposite for clinical use. Increased hydrophilicity and hardness with absorbability within the normal range can explain the reduced microorganism growth on the denture base, as has been proven in a previous study.

## 1. Introduction

With the aging of the population, the demand for prosthetic treatment in the stomatognathic system is growing [1]. In toothlessness or multiple teeth deficiencies, removable appliances with an extensive base are still the most common prosthetic solution. The force released in the act of chewing is completely passed by the prosthesis base onto the mucous membrane, the periosteum and bone. This creates the need for taking over by mucosa non-physiological functions, which results in its impaired physiology and increased susceptibility to infection. From the clinical point of view, polymethyl methacrylate (PMMA) has acceptable mechanical properties, nevertheless attempts have still been made to modify the material so that it would become resistant to microbial adhesion.

Some very promising nanoparticles (NPs) that can be used to modify the biomaterial for denture bases are zinc oxide (ZnO) NPs. Zinc oxide is a multi-functional material being a II-VI semiconductor [2]. Properties such as wide band gap (~3.37 eV) or high exciton binding energy (~60 meV) make it an attractive material for electronics and optoelectronics [3,4]. Selective doping of ZnO (NPs, alloys) with ions, for example, Cd^2+^, Co^2+^, Mn^2+^ permits changing its optical, magnetic and antibacterial properties, conductivity and photoluminescence [5,6,7,8]. The development of methods for obtaining nano-ZnO with a controlled shape and size enabled the commencement of research on the use of ZnO nanomaterials for the production of solar cells, gas sensors, switching memory devices and for water filtration [9]. In industry, ZnO is widely used as an ingredient of rubber, pigments, cements, plastics, sealants and paints. It is a component of pharmaceutical formulations and cosmetics, for example, baby powders, toothpastes and tooth dressings, sunscreens and skin protection balms. The advantages of ZnO-NPs application as a ultraviolet (UV) absorber in the field of personal hygiene and sun protection include long-term protection, broadband protection (UVA and UVB) and non-whitening effect on the skin [9,10,11]. It is also used in deodorants, medical and sanitary materials, glass, ceramic and self-cleaning materials [10,11]. The antibacterial and antifungal activity of ZnO-NPs was the major rationale for commencing in 2014 the research on dentures modified by ZnO-NPs, which are supposed to ultimately be characterized by antifungal properties [12,13]. At present, this issue has gained a growing interest among the scientist around the world and has already been investigated by several research groups [14,15,16,17,18,19,20].

The intention of our research group was to create new biomaterial for denture bases that could impede the adhesion of microorganisms to their surface, thereby decreasing development of denture stomatitis. It is of great importance for patients with immune system deficiencies (e.g., immunotherapy, AIDS, old age) where local fungal inflammation can lead to pneumonia or systemic fungemia endangering the patient’s life [21]. To achieve this goal, the modification of polymethyl methacrylate with zinc oxide nanoparticles was performed. The first article in a series [12] about new ZnO-PMMA nanocomposites presented the characteristics of ZnO-NPs (mean particle size, density, specific surface area). The minimal inhibitory concentration for standard strain of *Candida albicans* was determined at the level of 0.75 mg/mL. There, the process of performing nanocomposite was also described and the encountered difficulties presented in uniform distribution of ZnO-NPs in polymer matrix. Nevertheless, the sonication process decreased ZnO-NPs conglomerates, providing more favourable conditions for more homogenic material. Moreover, the SEM taken scans at a magnification of 50 k × confirmed that the material also contained particles smaller than 100 nm meeting the conditions required for nanomaterials. To our best knowledge this was the first successful attempt to produce PMMA resin for bases of dentures modified with nanoparticles of zinc oxide. The second publication [13] discussed the anti-fungal properties of newly formed biomaterial. These studies evidenced the antifungal activity of PMMA-ZnO nanocomposites. The study of the biofilm deposition on the surface showed that antifungal properties increase with increasing concentration of ZnO-NPs. A 7.5% nanocomposite revealed the lowest total amount of *Candida*, a 4.6-fold lower than in PMMA without modification. The (2,3-Bis-(2-Methoxy-4-Nitro-5-Sulfophenyl)-2H-Tetrazolium-5-Carboxanilide)(XTT) assay, in conjunction with testing the turbidity of solutions, may indicate the mechanism by which ZnO-NPs exert their effect on the increased induction of antioxidative stress in microorganism cells. The increased production of reactive oxygen species (ROS) can cause fungicidal action. The aim of this study was to investigate selected properties of nanocomposites, which can influence the microorganism deposition on their surface and better explain the results of the previous publication [13].

The basic property decreasing the deposition of food debris is the smoothness of the surface expressed by its roughness. A smaller expansion of the surface results in a reduced number of natural niches for *C. albicans*, which considerably hampers the formation of the fungal biofilm structure [22]. The hardness of the material does not directly affect the deposition of pathogens but becomes an exponent of the resistance supplement to mechanical damage [23]. It occurs during the act of chewing but also during the performance of denture hygiene especially when the patient, against medical advice, uses a substance with a high abrasion index. This can lead to the microdamage of prostheses and thereby increases the roughness parameter.

Another property that affects the deposition of microorganisms is hydrophobic or hydrophilic nature of the surface. With the increase in hydrophilic property the biofilm formation is reduced [24,25]. It is believed that the hydrophilic material interferes with the initial phase of microbial adhesion based on electrostatic interactions, Van der Waals forces and hydrogen interactions. *Candida* species have a very strong affinity to the surface of the acrylic base due to its specific nature but also because of its hydrophobic properties. In the cell wall of *Candida* there are adhesins, mainly mannoproteins, responsible for adhesion to host cells. Formosa et al. [26] have shown that depending on the conditions fungal cells can produce adhesins on their surface. Then they change the conformation of structures known as nanodomains, exhibiting hydrophobic properties and giving fungi affinity to all hydrophobic, abiotic surfaces, including acrylic resin material.

Absorbability of the material is also its negative feature. In the first stage of denture use the material absorbs fluids from the oral cavity to its interior. This results in an increase in weight of the material, which does not significantly affect the retention and function of the prosthesis but the liquid absorbed into the interior comprises microorganisms present in the saliva making formation of the fungal biofilm possible [27]. However, some authors [28] do not notice the correlation between the sorption of water and ease of accumulation of microorganisms. The PMMA porosity, regarded as one of the major causes of the biofilm formation, seems to be of minor importance. Studies conducted by Osica et al. [29] on the basis of computed tomography revealed an average porosity of PMMA at 0.01%. However, it should be remembered that this parameter varies over time, depending on the length of the prosthesis use and at a later stage it can play a role in the deposition of microorganisms. The process of biofilm formation and the mechanisms responsible for this process are multi-faceted and are the subject of interest to scientists all around the world. In previous articles, we characterized new material [12] and described antifungal properties of ZnO-PMMA nanocomposite [13]. The aim of this study was to investigate selected properties of ZnO-PMMA nanocomposites which can influence the microorganism deposition on their surface.

## 2. Materials and Methods

### 2.1. Synthesis and Characteristics of ZnO-NPs

ZnO-NPs were obtained using our microwave solvothermal synthesis method that permits a precise size control of the formed ZnO-NPs [12,13,30,31,32,33]. This method is characterized by the repeatability and reproducibility of properties of the synthesised ZnO-NPs. The following characteristics were defined for the obtained nanoparticles: morphology, skeleton density, specific surface area (SSA), phase purity and average particle size. The test procedures and the analysers employed in the characterization of ZnO-NPs used in this study were described extensively in our earlier publications [12,31,33]. The obtained powder had the following parameters: skeleton density 5.24 ± 0.05 g/cm^3^, specific surface area 39 ± 1 m^2^/g, average particle size (from SSA) 30 ± 0.1, average crystallite size (from Scherrer’s formula) 22–25 nm. ZnO-NPs were composed of single spherical crystallites. The ZnO powder contained only the hexagonal phase of ZnO (JCPDS card No. 36-1451 [30]).

### 2.2. Nanocomposite Preparation

A procedure of ZnO-PMMA nanocomposite preparation was described in a previously published article [12]. The main steps are described below. A thermally polymerized PMMA resin Superacryl Plus (Spofa Dental, Jiczyn, Czech Republic) was used to manufacture the samples. The recommended mixing ratio was 22 g of powder polymer and 10 mL of liquid monomer, which represents a volume ratio of 3:1. The appropriate amount of solvothermal zinc oxide nanopowder was suspended in liquid monomer of PMMA resin. The mixture was shaken in a Vortex VX-200 shaker (Labnet, Edison, NJ, USA) for 10 min and additionally sonicated for 240 s using an Elmasonic S 10/(H) (30 W, Elma Schmidbauer GmbH, Singen, Germany). Then a calculated amount of PMMA resin powder was added, so that the final 2.5%, 5% and 7.5% mass concentration of ZnO-NPs could be obtained. PMMA resin without ZnO-NPs was used as the control group.

In all the studies described below 3 types of nanocomposites (2.5%, 5% and 7.5%) were used and pure PMMA served as the control group.

### 2.3. Roughness Assay

The surface roughness was examined using a stylus profiler Dektak XT (Bruker, Billerica, MA, USA). Linear and aerial measurements were made. The former measures were taken in a single line and the latter measures in the area of the sample surface. Linear scans, 5 mm long, were taken step by step every 1 mm for both reference and modified samples. Moreover, topography of nanocomposites surface was imaged by colour three-dimensional (3D) laser microscope (VK-9700K, Keyence, Osaka, Japan). The surface area with dimensions of 1416 × 1000 μm was pictured. Examination was performed on 22 samples for each group of material. The data were evaluated for normal distribution using the Kolmogorov-Smirnov assay. Then after checking the homogeneity of variance (Brown-Forsythe assay) one test either Student’s *t*-test for independent samples (1) or Cochran Cox with separate variance estimate test (2) was performed. The level of significance was established at a *p*-value = 0.05. All data were computed using the Statistica 10.0 program (StatSoft Inc., Tulsa, OK, USA).

### 2.4. Contact Angle Assay

The hydrophobic/hydrophilic nature of the material before and after modification was determined by the contact angle. The value of the contact angle θ was determined by measuring the angle between the tested flat surface and the tangent created by a drop of liquid bordering with that body. Measurements were made at room temperature using a goniometer type ContactAngle^®^ system (OCA DeltaPhysics, Filderstadt, Germany). As a measuring liquid, distilled water was used. The drop volume measurement was 1 mL, dosing rate was equal to 2 mL/s. Examination was performed on 46 samples for each group of material. The data were evaluated for normal distribution using the Kolmogorov-Smirnov test. Then after checking the homogeneity of variance (Brown-Forsythe and Levene assays) one of the tests: *t*-Student for independent samples (1) or Cochran Cox with separate variance estimate test (2) was performed. The level of significance was established at a *p*-value = 0.05. All data were computed using the Statistica 10.0 program (StatSoft Inc., Tulsa, OK, USA).

### 2.5. Absorbability Assay

The measurement method for the water absorbency was elaborated based on standard procedure described in ISO 62:2008. To measure the water adsorption the samples with diameter of 20 mm × 20 mm × 2 mm were placed in liquid medium in a closed container. The chosen solution to perform this study was Fusayama’s artificial saliva, which mimics the oral environment. The Fusayama artificial saliva was prepared with the following composition: 0.2 g NaCl, 0.2 g KCl, 0.453 g CaCl_2_·2H_2_O, 0.345 g NaH_2_PO_4_·2H_2_O, 0.0025 g Na_2_S·9H_2_O, 0.5 g urea in 1000 mL of deionized water (pH 7). All chemicals were of analytical grade and used without further purification. All solutions were prepared with Milli Q water purified in the Millipore system. The samples were incubated at a human body temperature (37 °C). Subsequently, the samples weight changes were measured at a certain period of time (after 3, 5 and 7 weeks) utilizing a digital balance after slightly drying with a paper towel. Examination was performed on 24 samples for each group of material. The data were evaluated for normal distribution using the Kolmogorov-Smirnov test. Then after checking the homogeneity of variance (Brown-Forsythe and Levene assays) one of the tests: *t*-Student for independent samples (1) or Cochran Cox with separate variance estimate test (2) was performed. The level of significance was established at a *p*-value = 0.05. All data were computed using the Statistica 12.0 program (StatSoft Inc., Tulsa, OK, USA).

### 2.6. Hardness Assay

The hardness was examined according to ISO 868:2003 standard using a Shore durometer with scale D which is appropriate for the measurement of semi-rigid and hard plastics materials. The hardened steel rod configured as a needle pin (30° cone) under an applied force penetrates into the material and the depth of penetration is measured on a scale of 0 to 100. A given force of 44.64 N was applied in a consistent manner for a required period of time (15 s). All measurements were performed at room temperature. Examination was performed on 30 samples for each group of material. The data were evaluated for normal distribution using two independent assays, the Kolmogorov-Smirnov and Shapiro-Wilk tests. Then after checking the homogeneity of variance (Brown-Forsythe and Levene assays) one of the tests: *t*-Student for independent samples (1) or Cochran Cox with separate variance estimate test (2) was performed. The level of significance was established at a *p*-value = 0.05. All data were computed using the Statistica 12.0 program (StatSoft Inc., Tulsa, OK, USA).

## 3. Results and Discussion

### 3.1. Roughness Assay

**Figure 1:**
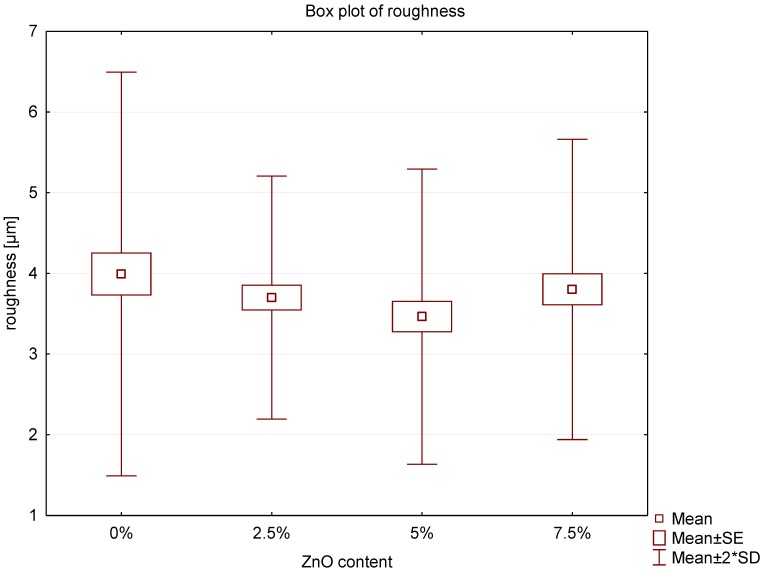
Box plot of the roughness of nanocomposites.

**Figure 2:**
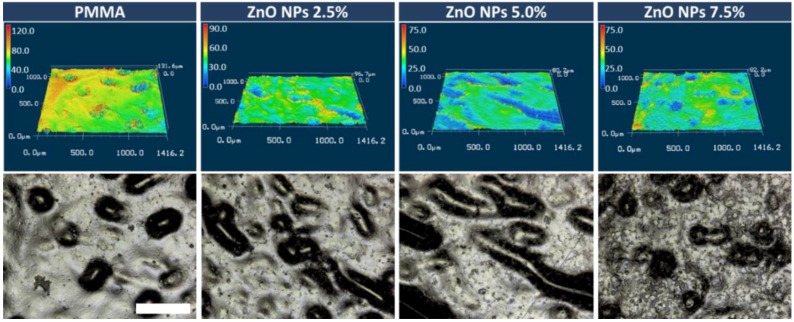
Surface topography of zinc oxide/polymethyl methacrylate (ZnO/PMMA) nanocomposites, the representative 3D laser microscope images (**top**) and optical laser-enhanced images of surfaces (**bottom**). The scale bar (white) refers to 400 µm and it is applicable to all images.

**Figure 3:**
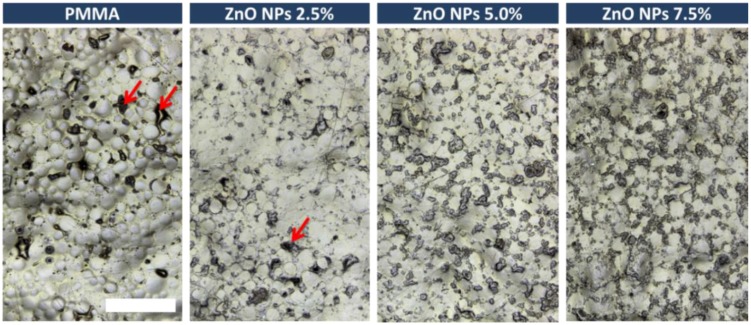
Optical laser-enhanced microscope images of the surfaces of ZnO/PMMA nanocomposites and pure PMMA for comparison. The scale bar (white) refers to 500 µm and it is applicable to all images. The red arrows mark the examples of surface cavities.

The study of the roughness of acrylic samples modified by the addition of ZnO-NPs are illustrated in box plot (Figure 1). The Ra parameter for control group was 3.99 µm with SD = 1.25. The results for 2.5%, 5% and 7.5% nanocomposites were: 3.70 µm (SD = 0.75); 3.46 µm (SD = 0.91) and 3.80 µm (SD = 0.93), respectively. Statistically significant differences were observed neither between 2.5%, 5% and 7.5% nanocomposites nor between nanocomposites and the control group. The three-dimensional surface imaging was obtained by 3D laser scans (Figure 2). The typical surface topography of the nanocomposites and pure PMMA was similar, characterized by large and deep grooves that were formed during the sample manufacture process. Depending on the content of ZnO nanopowder in nanocomposites a slight difference in surface smoothness could be observed (Figure 3), nevertheless it is not noticeable for roughness parameter measured on a micrometre scale.

There have been many studies showing positive correlation between the surface roughness and deposition of pathogenic microorganisms on the material’s surface [22,34,35]. The larger the development of the surface and the larger number of natural niches for microorganisms, the greater the potential for the deposition of *C. albicans.* These studies demonstrated neither positive nor negative effect of the addition of zinc oxide nanoparticles on the roughness parameter. The results are in line with the research carried out by Li et al. [36] who also found no statistically significant differences between PMMA and Ag-PMMA nanocomposite. The surface roughness of the samples averaged several micrometres, therefore, it seems unlikely that the additive particles in the nanometre scale could significantly affect the above parameter. Our study shows, however, that even in the same sample, wherein the average roughness is approximately 3 microns, the areas of high surface heterogeneity can be found (Figure 2 and Figure 3). Most likely, this is due to the manufacturing process of the prosthesis and the fact that it reflects of mucosal surface with all anatomic details (palatal folds or small salivary glands). It should be emphasized that the inner surface is not subjected to the polishing process. The prosthesis production procedure itself is not without significance, where the material is polymerized in gypsum form and can penetrate into the micropores of gypsum. Therefore, it is essential to use gypsum of high quality, adequate hardness and mixed in suitable proportions and even foil models to ensure acceptable smoothness of surface retaining all anatomical details. The roughness of the material is one of the key parameters affecting the deposition of microorganisms on the surface of PMMA and after modification of the material with zinc oxide nanoparticles this parameter does not change.

### 3.2. Contact Angle Assay

**Figure 4:**
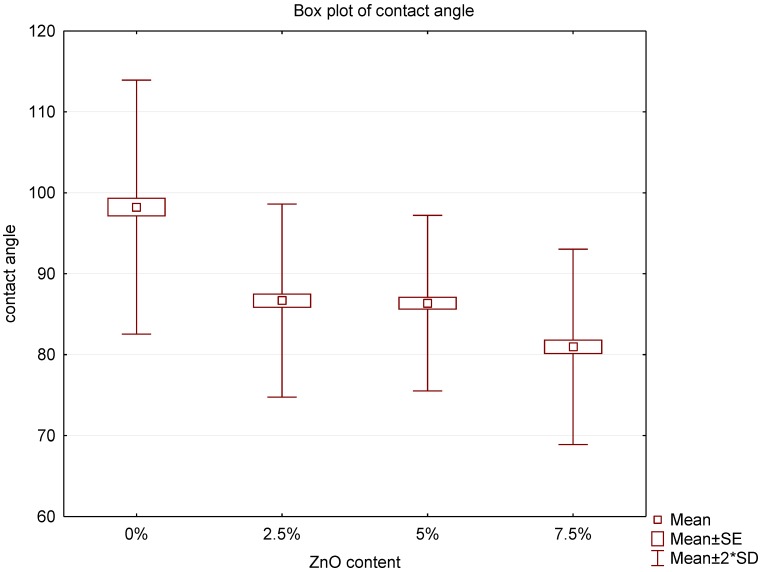
Box plot of the contact angle of nanocomposites.

The results of contact angle measurements are illustrated in a box plot (Figure 4). In all cases of nanocomposites, a statistically significant decrease in the value of the contact angle was observed compared to the control group. The contact angle parameter for the control group was 98.23 with SD = 7.85. Results for 2.5%, 5% and 7.5% nanocomposites were 86.67 (SD = 5.97); 86.36 (SD = 5.42) and 80.97 (SD = 6.03), respectively. No statistically significant differences between the 2.5% and 5% composites were observed, while in 7.5% nanocomposite the decline was at the level of 17.58% compared to the control group. This demonstrates the increased surface hydrophilicity after the addition of ZnO-NPs.

Hydrophobicity or hydrophilicity of materials is assessed on the basis of the contact angle measurement. The larger the angle between the biomaterial surface and liquid drops, the greater the hydrophobic properties exhibited by the biomaterial. The mechanism of microbial adhesion to the prosthesis base consists of two successive phases [37]. One reversible and non-specific uses, among others, electrostatic interaction and van der Waals forces. The other involves specific interactions between the adhesins present in the cell walls of microorganisms and the stereochemically complementar particles on the acrylic plate. *Candida* cells have on their surface hydrophobic polysaccharides, such as mannan or galactomannan, which interact not only with the epithelial mucosa but also with the surface of biomaterials [38]. In further stages of the biofilm formation the interactions between different species of microorganisms begin to play a greater role followed by a rapid growth of *C. albicans*. It has been shown that increasing hydrophilicity of the material hinders the process of microbial deposition [24,25]. Our present study shows that increasing amounts of ZnO-NPs in particular nanocomposites result in an increase in hydrophilic properties of the material. The interaction between water and ZnO nanoparticles leads to both molecular and dissociative adsorption of H_2_O forming a number of polar interactions and thus hydrophilic hydroxyl (–OH) species on the surface of particles [39]. The nanoparticles incorporated into the polymer matrix has a significant impact on the surface chemistry of the resulted nanocomposite, increasing its hydrophilicity. Therefore, original hydrophobic interactions between microorganisms and the denture base may be weakened. This finding has clinical implications, because the reduction of the adhesion of the first colonizing microorganisms can disrupt the formation of biofilm already in its initial phase and thus serve as a prophylactic agent for possible inflammation of the prosthetic base.

### 3.3. Absorbability Assay

**Figure 5:**
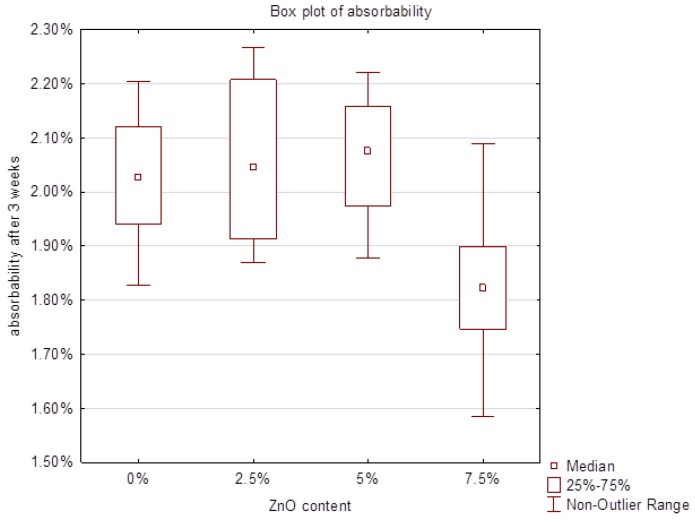
Box plot of the absorbability of nanocomposites after 3 weeks.

**Figure 6:**
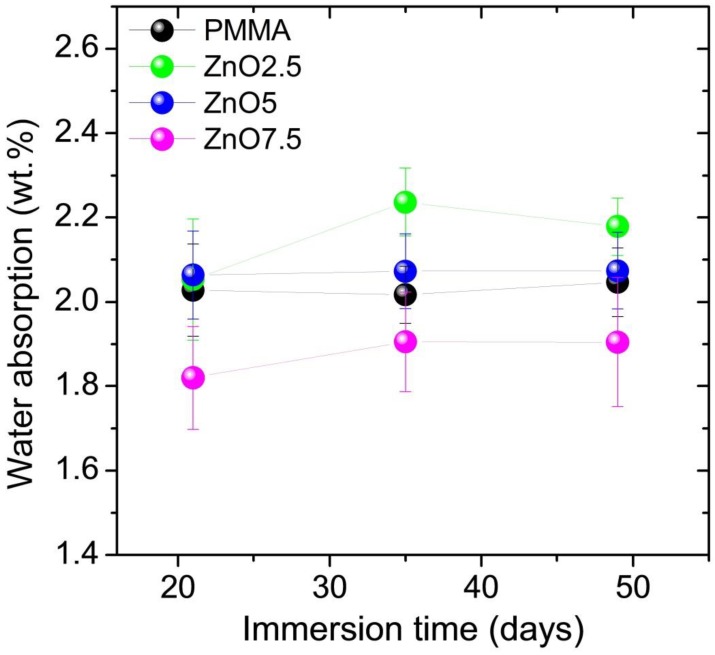
Changes in absorbability of materials after 3, 5 and 7 weeks.

The study of the absorbability of acrylic samples modified by the addition of ZnO-NPs after 3 weeks of incubation are illustrated in the box plot (Figure 5). No statistically significant differences were observed between the 2.5% or 5% nanocomposite and the control group, however the examination revealed statistically significant differences between the 7.5% nanocomposite compared to pure PMMA. All the results were within the range of 1.82–2.03%, which meets the requirements of ISO 20795-1:2013 Dentistry—Base polymers—Part 1: Denture base polymers norm. The lowest result was obtained in 7.5% nanocomposite and it was over 10% lower compared to control. Changes in absorption of examined materials, observed in two consecutive series, were small and after 7 weeks of incubation amounted from 0.13% for 2.5% nanocomposite to 0.01% for 5% nanocomposite (Figure 6). Finally, after 7 weeks the absorbability for the control group was 2.05% with SD = 0.08. The results for 2.5%, 5% and 7.5% nanocomposites were: 2.18% (SD = 0.07); 2.07 (SD = 0.09) and 1.90 (SD = 0.15), respectively.

Absorbability of materials specifies their possible absorption of liquids with suspended organic and inorganic components. In the oral environment, these components may include food debris and micro-organisms present in the saliva biocenosis. The higher the absorption, the higher the risk of colonization of biomaterial structures by pathogenic microorganisms and also the higher the probability of faster and less favourable changes in the colour and fragrance of the test material [27]. Water absorption by weight for acrylic resin used as denture bases averages 2% [40]. All tested materials showed absorption within the range of 1.82 to 2.03%, which meets the requirements of ISO 20795-1,2013 Dentistry—Base polymers—Part 1, Denture base polymers. The highest water sorption occurs during the first 3 weeks of incubation, update every 4 months was a maximum of 0.13%. The lowest water absorption was recorded for 7.5% nanocomposite (10% lower compared to the pure PMMA), which may contribute to the reduced colonization of microbes. Therefore, it could be expected that the base plate made of the above-mentioned biomaterial might be able to survive longer in the mouth, without changes in colour and smell.

### 3.4. Hardness Assay

**Figure 7:**
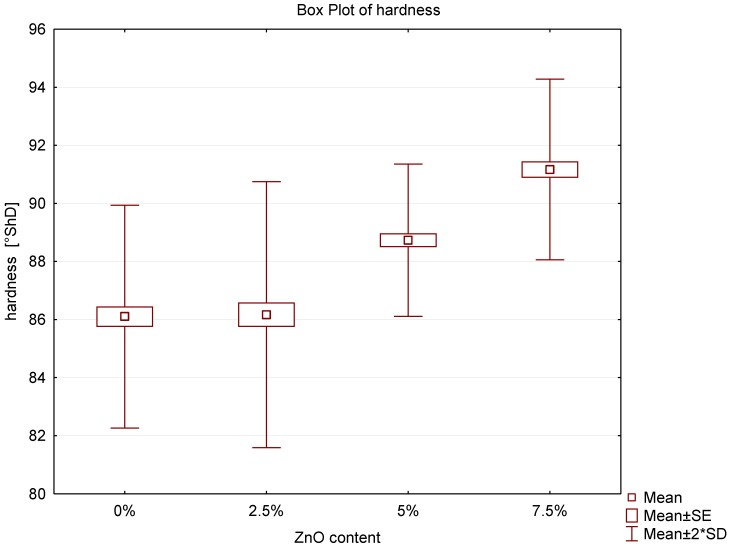
Box plot of the hardness of nanocomposites.

The study of the hardness of acrylic samples modified by the addition of ZnO-NPs are illustrated in the box plot (Figure 7). The determined hardness for the control group was 86.1° ShD with SD = 1.92. The results for 2.5%, 5% and 7.5% nanocomposites were 86.2° ShD (SD = 2.29); 88.7° ShD (SD = 1.31) and 91.2° ShD (SD = 1.56), respectively. No statistically significant differences were observed between the 2.5% nanocomposite and the control group, however the examination revealed statistically significant differences between 5% and 7.5% nanocomposites compared to pure PMMA. Moreover, the highest result was obtained for 7.5% nanocomposite and it was over 5.9% higher compared to control.

The hardness of the material is not directly linked with the deposition of fungal biofilm on the nanocomposite surface. Nevertheless, a possible use of this biomaterial as a dental prosthesis and the prevailing conditions in the oral cavity in which it will be used should be taken into account. It is essential to subject the denture to hygiene treatment after each meal to initiate the effect of mechanical and chemical stimuli. Patients, often against the doctor’s advice, use for denture cleaning a toothpaste with a high abrasion index containing unfavourable for PMMA abrasives that may scratch the surface of the denture. Any damage to denture, such as micro-scratches and cracks, caused during hygiene procedures or during chewing, may be natural niches for living microbes, including the most frequently isolated *C. albicans* in denture stomatitis. The hardness of the material can thus be the exponent of the prosthesis wear resistance capability [41]. According to theoretical considerations the addition of inorganic nanofiller particles to the polymer matrix increases the hardness of the material [42]. The results of this study show that the incorporation of ZnO-NPs to PMMA for 7.5% nanocomposite increases the hardness by 5.92% and this is a statistically significant difference. The revealed increase may hinder the formation of scratches and microcracks on the surface of the prosthesis which explains the reduced deposition of fungal biofilm on the surface of new biomaterials demonstrated in previous publications.

Moreover, the measurement of polymer hardness has been used successfully as an indirect method to assess the degree of conversion of the polymer in acrylic materials conventionally hot polymerized, as well as in composites [43,44]. The parameter of the material hardness is therefore sensitive to the increased content of residual monomer [45], one of the causes of allergic reactions and inflammation of mucosa under the denture base. Based on the presented results it can be concluded that in the entire study group the addition of ZnO-NPs to the polymer matrix does not contribute to the reduced hardness of plastic and thereby to the deteriorated degree of the polymer conversion. This observation is of extreme importance since the increased contents of residual monomer could influence not only the tendency to develop inflammation of the mucous membranes but also to exert a negative effect on mechanical properties of a new biomaterial.

## 4. Conclusions

This study is a continuation and extension of a previously published work [13] where anti-fungal properties of ZnO-PMMA nanocomposites, were demonstrated and the fact that they increase with increasing content of these zinc oxide nanoparticles was highlighted. The aim of the work was to investigate some properties of new biomaterials that could explain the origin of these properties. The obtained results of laboratory tests are encouraging. However, they must be clinically validated. It would be now interesting to carry out research to find out whether or not the addition of ZnO-NPs contributes at the same time to deterioration of the material mechanical properties. Therefore, prior to clinical application of the new biomaterial further mechanical and cytotoxic studies are required. Finally, it should be noted that the study showed no significant deterioration in the properties of acrylic resin, which could disqualify nanocomposite for clinical use. The increased hydrophilicity and hardness with absorbability within the normal range can explain the reduced microorganisms’ growth on the denture base what has been proven in a previous work. The presented study brings much closer the possibility of clinical use of new nanobiomaterials.
